# Rapid Antifungal Susceptibility Testing of Yeasts and Molds by MALDI-TOF MS: A Systematic Review and Meta-Analysis

**DOI:** 10.3390/jof7010063

**Published:** 2021-01-18

**Authors:** Miriam Alisa Knoll, Hanno Ulmer, Cornelia Lass-Flörl

**Affiliations:** 1 Institute of Hygiene and Medical Microbiology, Medical University of Innsbruck, 6020 Innsbruck, Austria; cornelia.lass-floerl@i-med.ac.at; 2 Department of Medical Statistics, Informatics and Health Economics, Medical University of Innsbruck, 6020 Innsbruck, Austria; hanno.ulmer@i-med.ac.at

**Keywords:** antifungal susceptibility testing, MALDI-TOF mass spectrometry, rapid AFST, detection of antifungal resistance

## Abstract

Due to the growing burden of fungal infections and a recent rise in antifungal resistance, antifungal susceptibility testing (AFST) is of increasing importance. The common methods of AFST have turnaround times of 24 to 48 h, and the available rapid methods are limited by applicability, cost-efficiency or accuracy. Given the urgency of adequate antifungal treatment in invasive mycoses, the need for the rapid and reliable detection of resistance is evident. In this systematic review and meta-analysis, we evaluated the diagnostic accuracy of AFST based on matrix-assisted laser desorption ionization time of flight mass spectrometry (MALDI-TOF MS). Twelve studies were reviewed, and data for the comparative analysis of their accuracy and methodology were systematically extracted. Compared to broth dilution as the gold standard, MALDI-TOF MS-based AFST reached a pooled sensitivity and specificity of 91% (95% Confidence Interval [CI], 84% to 96%) and 95% (95% CI, 90% to 98%), respectively. A comparative analysis showed that the sensitivity was higher for the semi-quantitative matrix-assisted laser desorption ionization Biotyper antibiotic susceptibility test rapid assay (MBT ASTRA) technique (96%) than for the correlate composite index (CCI) approach (85%), which is based on spectrum changes. Turnaround times below eight hours reached better diagnostic values than longer incubation periods, qualifying MALDI-TOF MS-based AFST as a rapid and accurate method for the detection of antifungal resistance.

## 1. Introduction

Fungal infections have been neglected in comparison to other infectious agents, despite their ubiquity and the rise of infections in the recent past. Worldwide, more than 1.5 million deaths per year are caused by fungal infections, which is comparable to the annual mortality rates of malaria and tuberculosis [1,2]. Advances in medicine have led to an increase of populations that are especially susceptible to fungal infections. In order to protect and treat those individuals, antifungal drugs are used in increasing amounts and for increasing durations, causing a selection of mutated fungal strains. Additionally, the extensive use of antifungals in agriculture enhances the development of antifungal resistance. Even with some new antifungal drugs in the pipeline, only five classes of antifungal drugs are available for patient treatment [3]. Due to intrinsic resistance of many fungal species and the limiting toxicities of some antifungals, the number of applicable drugs for fungal pathogens is even further narrowed. It is thus evident that any additional acquired resistance can have severe consequences for the affected individual, and the detection and surveillance of antifungal resistance are of increasing importance.

Antifungal susceptibility testing (AFST) is performed to investigate the response of a fungal pathogen to an antifungal drug. The gold standard for AFST is broth dilution, as specified by the current guidelines ((Clinical & Laboratory Standards Institute [CLSI] and European Committee on Antimicrobial Susceptibility Testing [EUCAST])). In order to perform those, 18 to 24 h cultures are required for EUCAST [4,5], while CLSI even requires double subculturing before testing a 24 h culture [6]. Before the results are available, broth dilution requires 24 to 48 h of incubation [4,5,6]. This long turnaround time represents a serious hindrance for the timely adjustment of an antifungal treatment and the initiation of disease control measures. The fact that broth dilution is still recommended as a reference method is due to the universal detection of resistance irrespective of the underlying mechanism. Fungal growth in the presence of an antifungal is the ultimate indicator for in vitro resistance, and visible growth quite simply takes time. Novel methods with shorter turnaround times have been developed, but all of those come with their own limitations of cost efficiency, applicability, or a limited number of detectable mechanisms [7,8,9].

Matrix-assisted laser desorption ionization time of flight mass spectrometry (MALDI-TOF MS) is a rapid, cost-effective and easy-to-handle method for microbial identification. This technology generates mass spectral profiles that are characteristic for each microorganism, which can be assigned to the respective species by comparison to a database. While it is well-established in microbial identification, attempts to utilize it in AFST have been relatively recent [10]. A variety of different approaches have been developed for the detection of resistance in bacteria [11], whereas AFST was investigated by merely two main approaches. The first method is based on the change of fungal spectrum profiles upon exposition to an antifungal substance. A Minimal Profile Change Concentration (MPCC) analogous to a minimum inhibitory concentration (MIC) value is inferred from a so-called composite correlation index (CCI) based on spectra from different concentrations of the antifungal substance. A second semi-quantitative technique named MBT ASTRA was originally developed for bacteria [12]. The growth of the infective agent is determined for different concentrations of antifungals and compared to a drug-free control, which provides a ratio of relative growth (RG). By defining a cut-off value, the RG at the corresponding antifungal concentration can be used to classify the quantity of the pathogen’s growth as susceptible or resistant. The turnaround times for those two approaches vary from three to thirty hours, with a median of six hours for the reviewed studies. 

As the time to adequate antifungal therapy is a major factor for patient outcomes in invasive mycoses [13], there is a demand for novel rapid technologies in AFST. In this systematic review and meta-analysis, we evaluate the existing data on MALDI-TOF MS applications in AFST, and assess the diagnostic accuracy of this method. We comparatively analyze the different technical approaches and evaluate the influence of test constellations as the tested species, antifungal substances, and incubation time on the performance of the assay. 

To our knowledge, this is the first analysis evaluating the diagnostic accuracy of MALDI-TOF MS applications in AFST, and the first study both to statistically compare the different approaches and to investigate their suitability for rapid diagnostic appliance.

## 2. Materials and Methods

### 2.1. Study Design

We performed a systematic review and meta-analysis of MALDI-TOF MS-based assays for the detection of antifungal resistance in yeasts and molds regarding azoles and echinocandins. The Preferred Reporting Items for Systematic Reviews and Meta-Analyses (PRISMA 2009) guidelines were used as the methodological support. The statistical analysis was conducted using MedCalc^®^ Statistical Software, version 19.5.3 (MedCalc Software Ltd., Ostend, Belgium; https://www.medcalc.org; 2020) and IBM SPSS Statistics for Windows, Version 25.0 (IBM Corporation, Armonk, NY, USA; 2019).

### 2.2. Search Strategy

Original articles published in English up to end of September 2020 were searched with PubMed and Google Scholar. ‘MALDI-TOF MS-based susceptibility testing’ and ‘mass spectrometry rapid detection resistance’ were combined with ‘fungi’, ‘Candida’ and ‘Aspergillus’. New links displayed beside the abstracts and articles mentioned in former reviews were followed. Finally, the bibliographies of each article were carefully reviewed, and relevant articles were also retrieved.

### 2.3. Inclusion and Exclusion Criteria

Only studies that evaluated antifungal susceptibility testing or the rapid detection of resistance towards antifungals through the use of MALDI-TOF MS technology were included. Additionally, the study reports must have had extractable data to fill the 4 cells of a 2 × 2 table for diagnostic tests (true resistant, false resistant, false susceptible and true susceptible). Additionally, broth dilution according to CLSI or EUCAST had to be used as reference standard or, if sequencing was originally mentioned as a gold standard, the data obtained by broth dilution had to be extractable.

### 2.4. Data Extraction

The full texts of the included articles were retrieved from the university library. The data from study reports was extracted twice. In order to ensure the reproducibility and completeness of the data extraction, an Excel spreadsheet (Microsoft Corp.) compiling all of the variables to be extracted was used. The data items included the authors, year of publication, tested antifungal drugs, technical approach, incubation time, sample size, reference method for broth dilution, sources of breakpoints or epidemiological cut-off (ECOFF) values, and values of true resistant, false resistant, false susceptible and true susceptible. When more than one antifungal or species was tested, these data were extracted separately. If possible, the raw data were used for the verification of the results. Otherwise, data for a 2 × 2 table for diagnostic tests and comparisons of agreement were extracted from the main text or the results section of the respective report. Strains classified as dose-dependently or intermediately susceptible by CLSI or EUCAST broth dilution were included in the calculations for essential and categorical agreement, but excluded from the preparation of the 2 × 2 tables. When more than one antifungal or species was tested, these data were extracted separately.

### 2.5. Breakpoints

The classification of the strains into susceptible (S), susceptible dose-dependent (SDD), intermediate (I) or resistant (R) was performed by applying the breakpoints (NCCLS, CLSI, EUCAST) or ECOFFs mentioned in the original report.

### 2.6. Data Analysis

Essential agreement was defined as ± 2 dilution steps around the MIC value obtained by broth microdilution. Categorical agreement was defined as concordant categorization (susceptible, susceptible dose-dependent, intermediate or resistant) by both broth dilution and MALDI-TOF MS. All of the results, irrespective of categorization according to EUCAST or CLSI broth dilution (S, SDD, I or R), were included in the analysis for essential and categorical agreement. 

For the comparative analysis, the studies were divided into groups based on their technical approach, antifungal substance, tested species, and incubation time. The sensitivity and specificity proportions of the selected studies were summarized using the meta-analysis for a proportion algorithm of the MedCalc^®^ Statistical Software Version 19.5.3 (MedCalc Software Ltd, Ostend, Belgium). This algorithm uses a Freeman–Tukey transformation (arcsine square root transformation; Freeman & Tukey, 1950 [14]) to calculate the weighted summary proportion under the fixed and random effects model (DerSimonian & Laird, 1986 [15]). Heterogeneity was significant in 69% of the sub-analyses. Therefore, the results were decided using the random effects approach. The results of the meta-analysis were visualized with forest plots. Sensitivity was defined as the proportion of drug resistant fungal strains correctly identified as resistant by MALDI-TOF MS. Specificity was defined as the proportion of susceptible strains correctly identified as susceptible by MALDI-TOF MS. Strains classified as susceptible dose-dependent or intermediate by CLSI or EUCAST broth dilution were excluded from the sensitivity, specificity and forest plots. An exception to this was Saracli et al., 2015 [16], in which the SDD for *C. glabrata* was defined as S, and thereby made comparable.

## 3. Results

**Figure 1:**
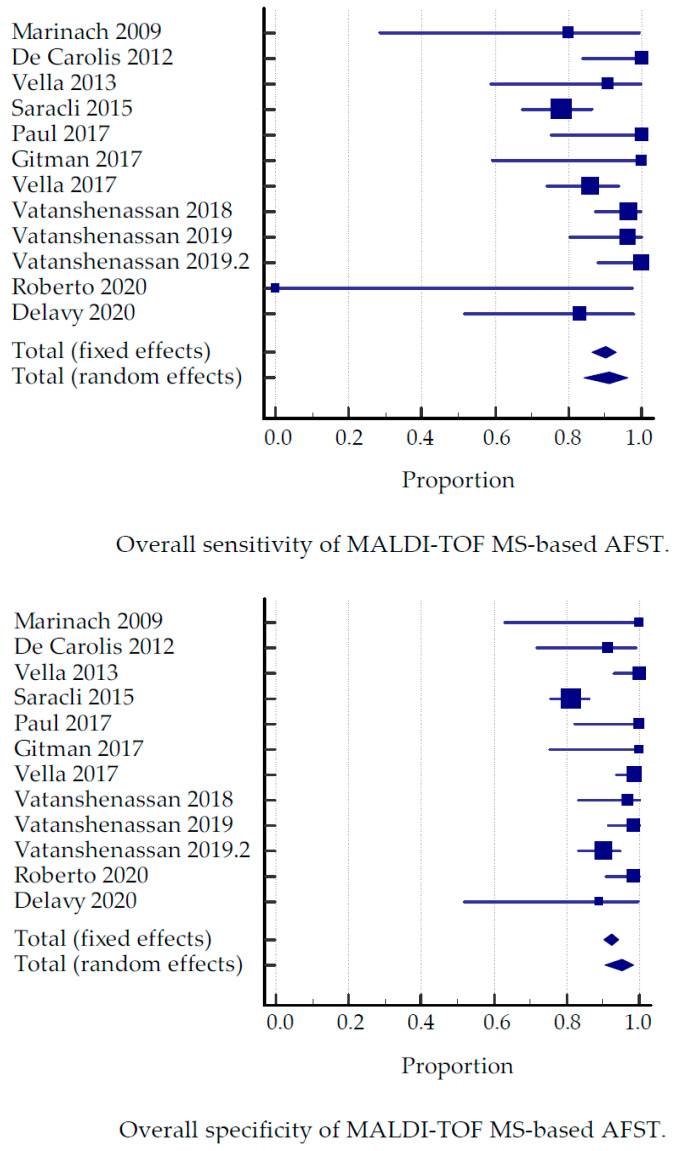
Overall sensitivity and specificity of MALDI-TOF MS-based AFST compared to the gold standard of broth dilution (CLSI or EUCAST).

In total, 424 articles were screened, and 17 full-text articles were assessed for eligibility. Twelve reports fulfilled the inclusion criteria for the meta-analysis. The study characteristics of the twelve included studies are summarized in Table 1. The collective sensitivity and 100-specificity for each study compared to the gold standard broth dilution (CLSI or EUCAST) are shown in Figure 1.

Overall, the MALDI-TOF MS-based detection of resistance achieved 91.1% sensitivity (95% Confidence Interval [CI], 84% to 96%) and 95.1% specificity (95% CI, 90% to 98%) compared to the gold standard broth dilution (CLSI or EUCAST).

### 3.1. Sub-Analysis by Approach

**Figure 2:**
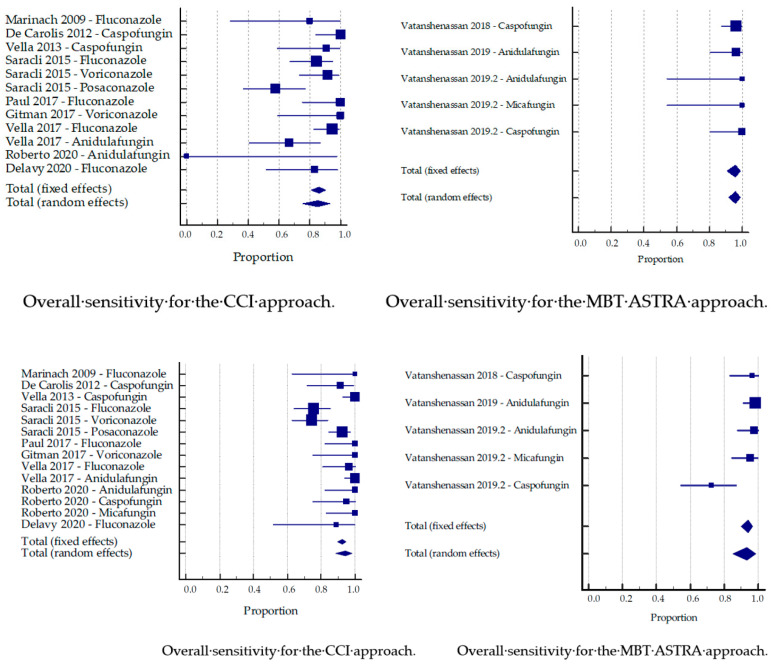
Sensitivity and specificity by technical approach.

The studies were divided into two groups based on their technical approach. Nine studies were based on the detection of altered spectrum profiles (CCI approach), and three studies were assigned to the MBT ASTRA technique. When more than one antifungal was tested, these study arms were analyzed separately. The overall sensitivity was higher for the MBT ASTRA approach, with 96% vs. 85.3% for the CCI approach, while the specificity was similar for both approaches (93.2% vs. 94.2%, respectively; Figure 2).

### 3.2. Sub-Analysis by Antifungal Substance

The studies were divided into two groups based on the tested antifungals. Six studies tested with azoles, and seven studies tested with echinocandins. When more than one antifungal was tested, these study arms were analyzed separately. 

The largest number of isolates was tested for caspofungin, with 104 resistant and 157 susceptible *Candida* and *Aspergillus* strains of different species. The highest sensitivity was reached for micafungin; however, only one study—with merely six resistant isolates—was suitable for analysis (Table 2). Micafungin also showed the best combined sensitivity and specificity. The highest specificity was achieved for anidulafungin. The lowest sensitivity was shown for posaconazole, and the lowest specificity was shown for voriconazole.

**Figure 3:**
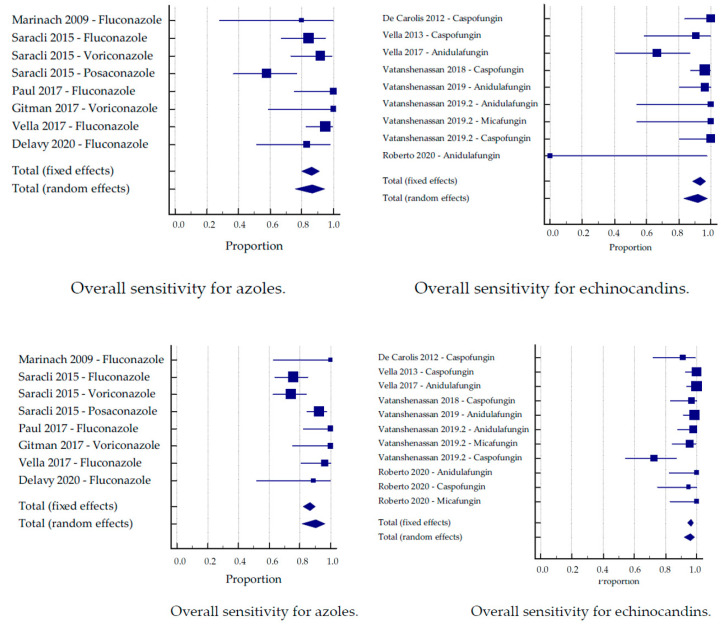
Sensitivity and specificity by the group of the antifungal substance.

Both the sensitivity and specificity were higher for echinocandins than for azoles, despite an outlier with a very low sensitivity for anidulafungin (Figure 3). The sensitivities for echinocandins and azoles were 92% and 86.4%, respectively. The specificities for echinocandins and azoles were 95.9% and 90.1%, respectively.

A subclassification into the two technical approaches was applicable for echinocandins. Again, the MBT ASTRA approach achieved better sensitivity than the CCI approach (96% and 93.2%, respectively), while better specificity was obtained by CCI (98.1%, compared to 79% for MBT ASTRA). Inversely, analyzing only studies based on the CCI approach, no significant difference in sensitivity between the two antifungal groups could be observed (84.4% for azoles vs. 83.3% for echinocandins). The specificity was still higher in the echinocandin group (97.3% vs. 92.3% for azoles).

### 3.3. Sub-Analysis by Species

The studies were divided into groups based on the tested species. When more than one antifungal was tested, these studies were analyzed separately. The largest number of samples per individual species was tested for *C. glabrata,* followed by *C. albicans*. Altogether, six studies analyzed *C. albicans*, five studies analyzed *C. glabrata*, five studies included other *Candida* species, and two studies analyzed different *Aspergillus* species.

A subclassification into the two approaches was applicable for *C. glabrata*. Again, the MBT ASTRA approach achieved a better sensitivity than the CCI approach (93.6% and 92.2%) and a lower specificity (83.9% and 92.6%, respectively).

**Figure 4:**
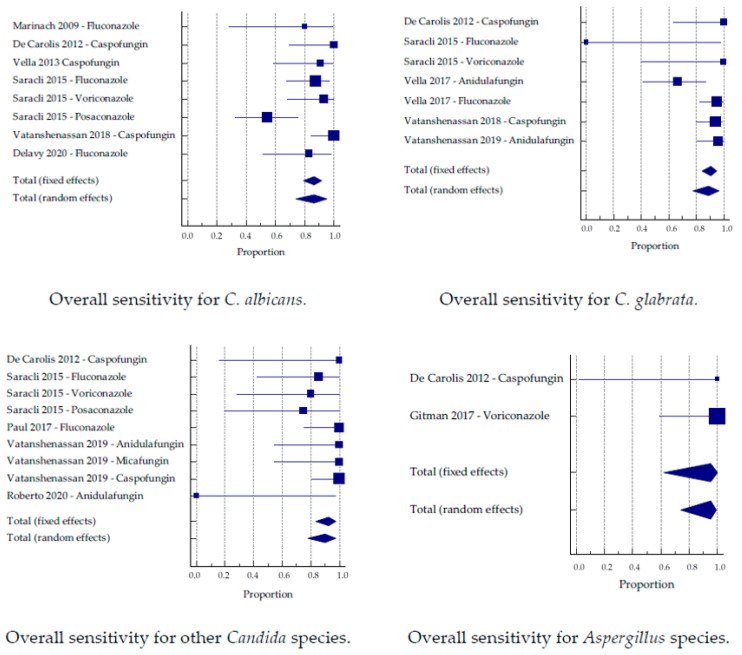
Sensitivity by the group of the antifungal substance.

**Figure 5:**
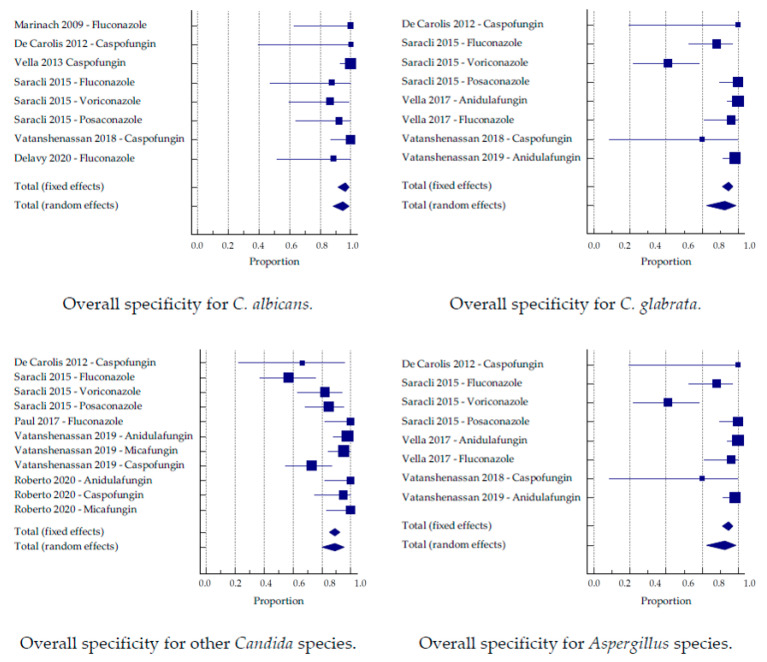
Specificity by the group of the antifungal substance.

The individually tested best sensitivity and specificity for yeasts was reached for *C. auris,* with 97.7% and 90%, respectively (Table 3). Altogether, 30 *Aspergillus* strains were included in the analysis, and both sensitivity and specificity were high (95.1% and 97.9%, respectively; Figure 4 and Figure 5).

### 3.4. Sub-Analysis by Time to Result

The studies were divided into groups based on the time to result. A cut-off of eight hours was defined to determine if the results were available within the same working day, and could thus be considered ‘rapid results’.

**Figure 6:**
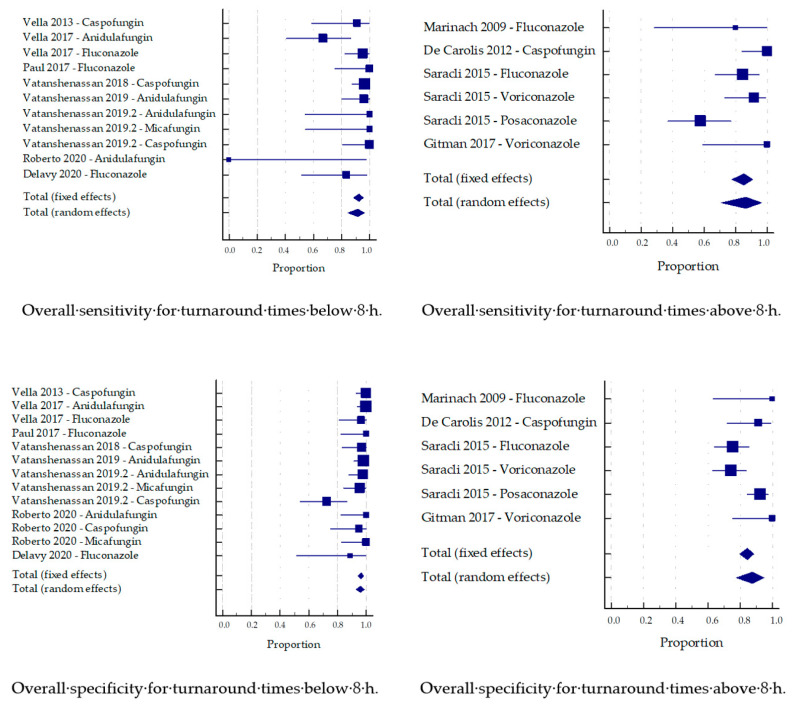
Sensitivity and specificity by turnaround time.

The pooled sensitivity and specificity for the turnaround times below eight hours were 91.4% and 96.1%, respectively, while the times above eight ours were both noticeably lower (86.3% and 87.5%; Figure 6). Analyzing only studies based on the CCI approach, still better specificity was achieved below eight hours (98.2% vs. 87.5% above eight hours), while this difference became less marked regarding sensitivity (87.5% below vs. 86.3% above eight hours).

## 4. Discussion

In invasive mycoses, early antifungal therapy is crucial for patient survival [13]. Inadequate antifungal therapy—and even short delays to the initiation of an adequate therapy—increase mortality rates in patients with candidemia [39,40,41]. Given the recent rise in the drug resistance of fungal pathogens, antifungal susceptibility testing (AFST) is becoming increasingly relevant [42,43,44,45,46,47,48]. As the common methods of AFST have turnaround times of 24 to 48 h, new rapid methods to detect resistances are necessary in order to ensure the fast and adequate adaption of antifungal therapy when needed.

The current methods for the rapid detection of resistance include molecular-based analyses [49,50], flow cytometry [51] or porous aluminum oxide (PAO)-based cultures [52,53], which deliver results within a few hours. Furthermore, the improvement of the conventional methods by the direct inoculation of standard tests with positive blood cultures [54] or shortening the time to result by automated approaches such as as the Vitek 2 yeast panel [55,56,57] have been investigated. However, all of these methods have their own limitations of applicability, diagnostic accuracy or time to result. MALDI-TOF MS has been investigated for application in AFST, as the technology is well established for species identification in routine microbiology. Many diagnostic laboratories are already equipped with a MALDI-TOF MS system, which would facilitate the utilization upon the development of an appropriate assay for AFST. In this meta-analysis, we provide an overview of the recent studies utilizing MALDI-TOF MS for AFST. The overall sensitivity and specificity of MALDI-TOF MS-based AFST has been evaluated at 91% and 95%, respectively. Given the further-expandable amount of data, these results suggest that MALDI-TOF MS is a promising new tool for rapid AFST.

Two main approaches for the utilization of MALDI-TOF MS in AFST have been established. The first method was developed in 2009 [17], and is based on a change of fungal spectrum profiles upon exposure to an antifungal substance. Spectra from different concentrations of the antifungal substance are used to create a composite correlation index (CCI) and thereby infer a Minimal Profile Change Concentration (MPCC) analogous to an MIC value. Some studies also evaluated a simplified three-point assay, in which fungal spectra at the breakpoint concentration were compared to a maximum concentration as well as a drug-free control, and were then classified into resistant or susceptible depending upon their similarity to one of the extremes [16,21,23,25,30]. However, while the hands-on time is somewhat shortened, the time to result mainly depends on the incubation time, and is thereby not abbreviated.

Originally developed for the rapid antibiotic susceptibility testing of bacteria, a second semi-quantitative technique named MBT ASTRA was recently adapted for AFST [33]. Instead of focusing on changes in the spectra of fungi, this method is based on the determination of the growth of cells in the absence or presence of an antifungal substance. The relative growth compared to a drug-free control is calculated for each concentration, and a cut-off is defined in order to classify the relative growth values as resistant or susceptible.

Comparing these two approaches, MBT ASTRA showed, overall, a better sensitivity in our meta-analysis. While some studies also obtained high diagnostic values with the CCI approach, these results were rather heterogeneous. In contrast, consistently high sensitivity was achieved by MBT ASTRA. In regard to specificity, no significant differences could be observed between the two approaches. Considering the other results of this meta-analysis, one could deduce that this difference in sensitivity may be caused by the choice of more suitable antifungal drugs and species for analysis. While Vatanshenassan et al. tested with echinocandins only, many of the CCI studies were performed using azoles, for which generally-worse diagnostic results were obtained. In order to evaluate whether the choice of the tested antifungal had an influence on these results, a sub-analysis divided by the approach was performed. Only studies using echinocandins were compared, and still a markedly-higher pooled sensitivity was obtained by MBT ASTRA than by CCI. The same trend could be shown upon direct comparison on a species level, in which—among studies testing *C. glabrata*—those using the MBT ASTRA technique obtained higher sensitivities than the CCI approach. In conclusion, the better diagnostic results for the semi-quantitative MBT ASTRA seem to be partly due to a better selection of the tested bug–drug combinations, but upon close examination of the data, the measure of relative growth seems to obtain better results than the comparison of spectra, independently of the test conditions.

Upon division into groups based on the antifungal substance, better diagnostic values for echinocandins become apparent. However, only one study tested members of both antifungal classes, thereby enabling a direct comparison by the same setup of experiments [23]. In this study, no clear difference between the antifungals is observable. In order to exclude a bias by the implemented technical approach, only studies working with the CCI technique were compared. In doing so, no significant difference in sensitivity between the two antifungal groups could be observed, whilst the specificity was still better for echinocandins. Not enough data were available for the performance of this sub-analysis on MBT ASTRA-based studies. Given that the CCI approach is based on spectrum changes, those differences might be due to a different influence on the proteome by the respective antifungal. However, since MALDI-TOF MS-based AFST is fairly new, this is—to our knowledge—the first report on different diagnostic values between antifungal classes. In conclusion, the overall analysis favors diagnostic results for echinocandins over azoles, but the reason might be partly due to a bias in the technical approach. More data with direct comparisons of two different classes of antifungal substances would be necessary. Nevertheless, the goal of developing new methods for rapid AFST should be to obtain valid and reliable results regardless of species or antifungal substance.

Similarly, upon the separation of the studies on a species level, differences in sensitivity and specificity between different yeast and mold species can be recognized. However, the only species with an overall case number above 100 tested isolates were *C. albicans* and *glabrata*. These species have been tested in several studies, and an overall sensitivity of 86.6% for *C. albicans* and 88.8% for *C. glabrata* was obtained. The specificity was above 90% for both species, which was lower than the overall values (91% sensitivity, 95% specificity). Good results were obtained for *Aspergilli,* which were included in two studies [21,27]. While AFST for molds commonly requires at least 24–48 h, De Carolis et al. were able to obtain a sensitivity and specificity of 100% within merely 15 h for different *Aspergilli*. In a second study, the incubation time was prolonged to 30 h for valid results [21], which still represents a reduction of the time to result compared to common methods. Only a small number of isolates has been tested, and not enough data is available for a conclusion, but nevertheless, it appears to be a promising tool for rapid AFST of *Aspergillus* sp. For other species, so far, not enough data is available to reliably compare the diagnostic accuracy. Hence, the very high or low diagnostic accuracies in this meta-analysis for some species have to be attributed to the small number of tested isolates, and are therefore not comparable. We have to assume that those values will approximate to the general values.

The eventual objective of the development of new methods for AFST is to obtain reliable results within the shortest possible time. However, shortening the time to result should not be traded against accuracy, but rather reach the same reliability as the reference method. Since this is a balancing act, one would suppose that shorter incubation times naturally come with lower diagnostic accuracy. Remarkably, in this meta-analysis, turnaround times below 8 h achieved better pooled sensitivity and specificity compared to studies with incubation times above 8 h. One confounding factor could be the distribution of the two technical approaches, since all of the studies based on MBT ASTRA with better sensitivity than CCI based reports were in the group with shorter incubation times. Analyzing only the CCI based studies, the difference becomes smaller, and only the specificity remains markedly better in the group of shorter times to result. This could be due to the fact that newer studies tended to apply shorter incubation times, and that expertise and technical know-how have increased over the years. Nonetheless, it is substantial to note that MALDI-TOF MS-based AFST can achieve satisfactory diagnostic results in under 8 h, which qualifies the method for future rapid applications in routine diagnostics.

In summary, the target of MALDI-TOF MS-based AFST is the rapid and reliable detection of resistance, in order to ensure a prompt modification of therapy if it is proven necessary. Vatanshenassan et al. [35] recently evaluated AFST via MALDI-TOF MS directly from positive blood cultures, which will presumably become one of the future adaptions to investigate. The reviewed articles cover a highly-divergent setup of experiments with different fungal species, antimycotics, microdilution methods and breakpoints. Despite this limitation, some conclusions can be drawn from the currently-available literature. Given the results of this analysis, reaching short incubation times without decreasing diagnostic accuracy is feasible. Better sensitivity has been achieved by the application of the semi-quantitative MBT ASTRA technique compared to the CCI approach based on spectrum changes. With a sensitivity of 91% and specificity of 95% overall, MALDI-TOF MS-based AFST has proven to be a promising new method for the rapid detection of resistance in fungi. Further investigations using a more homogenous methodology with a larger number of isolates from a range of species and antifungal drugs will be necessary.

## Figures and Tables

**Table 1:** Characteristics of the included studies (*n* = 12).

	Antifungal	Species	Incubation Time	Essential Agreement	Categorical Agreement	Sensitivity	95% CI	Specificity	95% CI	True Resistant	False Resistant	True Susceptible	False Susceptible	Reference Method/Breakpoints
*CCI Approach*														
Azoles														
Marinach 2009 [17]	Fluconazole	*C. albicans*	15 h	100%	88%	80%	28% to 99%	100%	63% to 100%	4	0	8	1	CLSI M27-A2 [18]
Saracli 2015 [16]	Fluconazole	*C. albicans*	16 h	n.a.	80%	86% *	68% to 97%	88% *	47% to 100%	21	1	7	3	CLSI M27-A3 [19]
		*C. glabrata*	16 h	n.a.	86%	0%	0% to 98%	88% *	73% to 97%	0	4	30	1	CLSI M27-A3 [19]
		*C. tropicalis*	16 h	n.a.	65%	86%	42% to 100%	57% *	37% to 76%	6	12	16	1	CLSI M27-A3 [19]
	Voriconazole	*C. albicans*	16 h	n.a.	91%	93% *	68% to 100%	87% *	60% to 98%	14	2	13	1	CLSI M27-A3 [19]
		*C. glabrata*	16 h	n.a.	66%	100%	40% to 100%	61% *	42% to 78%	4	12	19	0	CLSI M27-A3 [19]
		*C. tropicalis*	16 h	n.a.	84%	80% *	28% to 99%	82% *	63% to 94%	4	5	23	1	CLSI M27-A3 [19]
	Posaconazole	*C. albicans*	16 h	n.a.	69%	55% *	32% to 76%	92% *	64% to 100%	12	1	12	10	CLSI M27-A3 [19]
		*C. glabrata*	16 h	n.a.	100%	n.a.	n.a.	100% *	90% to 100%	0	0	35	0	CLSI M27-A3 [19]
		*C. tropicalis*	16 h	n.a.	84%	75% *	19% to 99%	85% *	68% to 95%	3	5	28	1	CLSI M27-A3 [19]
Paul 2017 [20]	Fluconazole	*C. tropicalis*	4 h	100%	94%	100%	75% to 100%	100%	82% to 100%	13	0	19	0	CLSI M27-A3 [19]
Gitman 2017 [21]	Voriconazole	*Aspergillus* spp.	30 h	100%	100%	100%	59% to 100%	100%	75% to 100%	7	0	13	0	Pfaller 2009 [22]
Vella 2017 [23]	Fluconazole	*C. glabrata*	3 h	n.a.	79%	95%	83% to 99%	96%	81% to 100%	37	1	26	2	CLSI M27-S4 [24]
Delavy 2020 [25]	Fluconazole **	*C. albicans*	3 h	86%	n.a.	83%	52% to 98%	89%	52% to 100%	10	1	8	2	EUCAST v.9 [26]
Echinocandins														
De Carolis 2012 [27]	Caspofungin	*C. albicans*	15 h	100%	100%	100%	69% to 100%	100%	40% to 100%	10	0	4	0	Pfaller 2011 [28]
		*C. glabrata*	15 h	100%	100%	100%	63% to 100%	100%	40% to 100%	8	0	4	0	Pfaller 2011 [28]
		*C. parapsilosis*	15 h	100%	100%	n.a.	n.a.	100%	40% to 100%	0	0	4	0	Pfaller 2011 [28]
		*C. krusei*	15 h	100%	50%	100%	16% to 100%	0%	0% to 84%	2	2	0	0	Pfaller 2011 [28]
		A. *fumigatus*	15 h	100%	100%	100%	3% to 100%	100%	48% to 100%	1	0	5	0	Espinel-Ingroff 2011 [29]
		A. *flavus*	15 h	100%	100%	n.a.	n.a.	100%	40% to 100%	0	0	4	0	Espinel-Ingroff 2011 [29]
Vella 2013 [30]	Caspofungin	*C. albicans*	3 h	n.a.	98%	91%	59% to 100%	100%	93% to 100%	10	0	50	1	Pfaller 2011 [28]
Vella 2017 [23]	Anidulafungin	*C. glabrata*	3 h	n.a.	88%	67%	41% to 87%	100%	94% to 100%	12	0	58	6	CLSI M27-S4 [24]
Roberto 2020 [31]	Anidulafungin	*C. parapsilosis complex*	3 h	90%	95%	0%	0% to 98%	100%	82% to 100%	0	0	19	1	CLSI M27-A3 & -S4 [19,24]
	Caspofungin	*C. parapsilosis complex*	3 h	95%	95%	n.a.	n.a.	95%	75% to 100%	0	1	19	0	CLSI M27-A3 & -S4 [19,24]
	Micafungin	*C. parapsilosis complex*	3 h	95%	100%	n.a.	n.a.	100%	83% to 100%	0	0	20	0	CLSI M27-A3 & -S4 [19,24]
*MBT ASTRA*														
Echinocandins														
Vatanshenassan 2018 [32]	Caspofungin	*C. albicans*	6 h	91%	100%	100%	85% to 100%	100%	87% to 100%	22	0	25	0	CLSI M60 [33]
		*C. glabrata*	6 h	93%	61%	94%	80% to 99%	80%	28% to 99%	31	1	4	2	CLSI M60 [33]
Vatanshenassan 2019 [34]	Anidulafungin	*C. glabrata*	6 h	90%	89%	96%	80% to 100%	98%	91% to 100%	25	1	62	1	CLSI M60 [33]
Vatanshenassan 2019 [35]	Anidulafungin	*C. auris*	6 h	80%	98%	100%	54% to 100%	98%	88% to 100%	6	1	43	0	Tentative BPs [36,37,38]
	Micafungin	*C. auris*	6 h	86%	96%	100%	54% to 100%	96%	85% to 99%	6	2	42	0	Tentative BPs [36,37,38]
	Caspofungin	*C. auris*	6 h	68%	82%	100%	80% to 100%	73%	54% to 87%	17	9	24	0	Tentative BPs [36,37,38]

* After the application of a 5% tolerance rate; ** ± Cyclosporine A; Abbreviations: CI: Confidence Interval; n.a.: not applicable; BPs: breakpoints.

**Table 2:** Sensitivity and specificity by antifungal agent.

Antifungal	Number of Resistant Isolates	Sensitivity (%)	Number of Susceptible Isolates	Specificity (%)
Fluconazole	101	89.3	133	91.4
Posaconazole	26	57.7 *	81	92.6 *
Voriconazole	31	92.0	87	88.0
Caspofungin	104	96.4	157	92.1
Anidulafungin	51	79.6	184	98.4
Micafungin	6	100 *	64	96.2

* Only one study was included in the analysis.

**Table 3:** Sensitivity and specificity by species.

Species	Number of Resistant Isolates	Sensitivity (%)	Number of Susceptible Isolates	Specificity (%)
*C. albicans*	121	86.6	133	94.7
*C. glabrata*	129	88.8	257	92.5
*C. tropicalis*	29	86.7	108	82.7
*C. parapsilosis*	1	0 *	63	97.2
*C. krusei*	2	100 *	2	0 *
*C. auris*	29	97.7 *	121	90 *
*Aspergillus fumigatus*	5	100	18	100
Other *Aspergillus* species	3	100 *	4	100 *

* Only one study was included in the analysis.
